# Oral-Nasopharyngeal Dendritic Cells Mediate T Cell-Independent IgA Class Switching on B-1 B Cells

**DOI:** 10.1371/journal.pone.0025396

**Published:** 2011-09-29

**Authors:** Kosuke Kataoka, Keiko Fujihashi, Yutaka Terao, Rebekah S. Gilbert, Shinichi Sekine, Ryoki Kobayashi, Yoshiko Fukuyama, Shigetada Kawabata, Kohtaro Fujihashi

**Affiliations:** 1 Department of Preventive Dentistry, Institute of Health Biosciences, The University of Tokushima Graduate School, Tokushima, Japan; 2 Departments of Pediatric Dentistry and Microbiology, The Immunobiology Vaccine Center, The University of Alabama, Birmingham, Alabama, United States of America; 3 Department of Oral and Molecular Microbiology, Graduate School of Dentistry, Osaka University, Osaka, Japan; 4 Department of Preventive Dentistry, Graduate School of Dentistry, Osaka University, Osaka, Japan; Albany Medical College, United States of America

## Abstract

Native cholera toxin (nCT) as a nasal adjuvant was shown to elicit increased levels of T-independent S-IgA antibody (Ab) responses through IL-5- IL-5 receptor interactions between CD4^+^ T cells and IgA^+^ B-1 B cells in murine submandibular glands (SMGs) and nasal passages (NPs). Here, we further investigate whether oral-nasopharyngeal dendritic cells (DCs) play a central role in the induction of B-1 B cell IgA class switch recombination (CSR) for the enhancement of T cell-independent (TI) mucosal S-IgA Ab responses. High expression levels of activation-induced cytidine deaminase, Iα-Cμ circulation transcripts and Iμ-Cα transcripts were seen on B-1 B cells purified from SMGs and NPs of both TCRβ^−/−^ mice and wild-type mice given nasal trinitrophenyl (TNP)-LPS plus nCT, than in the same tissues of mice given nCT or TNP-LPS alone. Further, DCs from SMGs, NPs and NALT of mice given nasal TNP-LPS plus nCT expressed significantly higher levels of a proliferation-inducing ligand (APRIL) than those in mice given TNP-LPS or nCT alone, whereas the B-1 B cells in SMGs and NPs showed elevated levels of transmembrane activator and calcium modulator cyclophilin ligand interactor (TACI) expression. Interestingly, high frequencies of IgA^+^ B-1 B cells were induced when peritoneal IgA^−^ IgM^+^ B cells were stimulated with mucosal DCs from mice given nasal TNP-LPS plus nCT. Taken together, these findings show that nasal nCT plays a key role in the enhancement of mucosal DC-mediated TI IgA CSR by B-1 B cells through their interactions with APRIL and TACI.

## Introduction

Immunoglobulin A (IgA) antibody (Ab) is known as the most abundant Ig isotype in humans, and this isotype is induced by cellular and molecular mucosal interactions between IgA-committed B cells, helper CD4^+^ T cells, epithelial cells and their derived cytokines. [1], [2] In order to induce efficient antigen (Ag)-specific IgA Ab responses, live attenuated viral or bacterial delivery systems or mucosal adjuvants are generally required. Mucosal adjuvants, including enterotoxins [3], [4], cytokines [5], [6], chemokines [7], toll-like receptor ligands [8] and growth factors [9] offer the advantage of eliciting mucosal as well as parenteral immune responses [10]. Of these, native cholera toxin (nCT) is the most widely used mucosal adjuvant for the induction of both mucosal and systemic immunity to co-administered protein Ags in mice. Thus, nasal administration of nCT as mucosal adjuvant preferentially induces characteristic plasma IgG1, IgG2b, IgE and IgA as well as mucosal S-IgA Ab responses to T cell-dependent (TD) Ags, which are mediated through CD4**^+^** Th2-type cells and their derived cytokines [3], [11]–[13].

Nasopharyngeal-associated lymphoreticular tissue (NALT) and Peyer's patches are specialized lymphoid tissue clusters known as mucosal inductive sites where IgA-committed B cells undergo μ to α isotype class switching. Subsequently, IgA-committed B cells migrate to diffuse mucosal effector tissues, including the nasal passages (NPs) and intestinal lamina propria (iLP), respectively [14], [15]. In addition to these mucosal inductive tissues, it is known that IgA CSR occurs in the absence of T cells in the iLP [16]–[18]. Similarly, B cells of the isolated lymphoid follicles (ILFs), scattered throughout the intestine, can undergo IgA CSR either from actual bacterial infection or from constant surveillance of commensals [19], [20]. In this regard, both *in vitro* and *in vivo* studies have shown that a proliferation inducing ligand (APRIL) promotes T cell-independent (TI) CSR of IgA via engagement of transmembrane activator and calcium modulator cyclophilin ligand interactor (TACI) [21]–[23]. Recent studies reported that retinoic acid-producing DCs from mucosa-associated lymphoreticular tissue induce surface IgA and gut homing receptor expression on B cells in a TI manner [24]. Since APRIL binds firmly to B-cell maturation antigen (BCMA) in addition to TACI, APRIL on DCs interacts with BCMA and TACI on B cells in order to induce IgA CSR [25].

B-1 B cells differ from conventional B cells in cell surface protein CD5 expression, anatomical localization and functional characteristics [26]–[28]. With regard to function, B-1 B cells differentiate primarily into Ab-producing plasma cells of all isotypes in response to polysaccharide Ag. Although these responses can be enhanced/increased by T cells, they appear within 48 hr of exposure to Ag and are not dependent upon T cell help. B-1 B cells in the murine peritoneal cavity and iLP have been shown to develop from a common pool and may represent a lineage separate from conventional PP B cells [29]. Other studies using transgenic mice have provided additional supportive evidence that intestinal IgA plasma cells are derived from B-1 B cells [30]. Furthermore, it has been shown that intestinal mucosal IgA Abs with specificity for commensal bacteria are produced by B-1 B cells in a TI manner [31], [32]. These studies clearly suggest that B-1 B cells are an important source of IgA-producing cells in the intestinal mucosal tissues.

Although IgA^+^ B cell isotype switching is known to occur in organized mucosal inductive lymphoid tissues such as PPs, NALT and ILFs [33], little information is available regarding the cellular and molecular mechanisms underlying IgA class switch recombination (CSR) to TI Ag in the oral and nasal mucosa. We have previously reported that nCT can be used as an adjuvant to enhance the induction of TI Ag, i.e., TNP-LPS-specific mucosal S-IgA Ab responses through an interaction between IL-5 receptor-expressing B-1 B cells and IL-5-producing CD4^+^ T cells in the SMGs and NPs [34]. In this study, we hypothesized that IgA switching occurs in B-1 B cells in the SMGs and NPs via the Ig isotype switching-associated molecules produced by mucosal DCs. Here we focused on the cellular and molecular mechanisms for the induction of IgA CSR induced by nasal nCT as a mucosal adjuvant for immune responses to TI Ags.

## Materials and Methods

### Mice and nasal immunization

Female C57BL/6 mice were purchased from SLC Japan (Tokyo, Japan) and were transferred to microisolators, maintained in horizontal laminar flow cabinets in the University of Tokushima animal facility. TCRβ-deficient (TCRβ^−/−^) mice were barrier maintained in Trexler isolators in the University of Alabama at Birmingham (UAB) animal facility. These mice were provided sterile food and water as part of a specific pathogen free facility. All of the mice were 8 to 12 wks old and free of bacterial and viral pathogens when used for these experiments. All mice were housed and used in accordance with the University of Tokushima and UAB guidelines for the care and use of laboratory animals. Mice were immunized nasally with 10 µg of TNP-LPS (Sigma Aldrich Co.) in the presence (experimental group) or absence (control group) of native CT (nCT, 1 µg) (List Biological Laboratories Inc.) under anesthesia. In some experiments, mice were given nasal PBS or nCT alone (1 µg).

### Isolation of mononuclear cells from mucosal tissues and enumeration of IgA-producing cells

Mononuclear cells were isolated from the submandibular glands (SMGs), nasal passages (NPs) and nasopharyngeal associated lymphoid tissues (NALT), as described previously. Briefly, mononuclear cells from SMGs were isolated by an enzymatic dissociation procedure with collagenase type IV (0.5 mg/ml; Sigma) followed by a discontinuous Percoll (GE Healthcare Bio-Sciences) gradient centrifugation step [34]. For isolation of NPs, a modified dissociation method based upon a previously described protocol was used [34], [35]. Mononuclear cells from NALT were obtained using a mechanical dissociation method that involved gentle teasing through stainless steel wire screens as described elsewhere [5], [34], [35]. In some experiments, mononuclear cells obtained from SMGs and NPs were subjected to an ELISPOT assay to detect numbers of TNP-specific IgA Ab-forming cells (AFCs). Briefly, 96-well nitrocellulose plates (Millititer HA; Millipore Corporation) were coated with 10 µg/ml TNP-BSA. The numbers of TNP-specific IgA AFCs were quantified with the aid of a stereomicroscope, as well as ImmunoSpot Analyzer reader (Cellular Technology Ltd.) as described elsewhere [34].

### Flow cytometric analyses and cell sorting

The mononuclear cells from various mucosal tissues were isolated 3 days after nasal immunization and preincubated with purified CD16 mAb (Fc Block, 2 µg/ml; BD PharMingen). Samples were then stained with PE-labeled anti-CD11c and purified anti- BAFF (5A8; rat IgG1) or anti-APRIL (Sacha-2; rat IgG2a) followed by FITC-conjugated anti-rat IgG1 or IgG2a mAbs (BD Biosciences). Aliquots of samples were also incubated with purified anti-BAFF receptor (BAFF-R) (7H22-E16; rat IgG1), anti-TACI (8F10; rat IgG2a) or anti-BCMA (Vicky-2; rat IgG2a) mAb followed by FITC-conjugated anti-rat IgG1 or IgG2a mAb. These samples were further incubated with allophycocyanin (APC)-conjugated anti-B220 and PE-labeled anti-CD5 followed by addition of 7-amino actinomycin D solution (Via-Probe; BD Biosciences) to exclude dead cells. BAFF and APRIL, as well as their receptor-specific mAbs originally from ALEXIS Biochemicals (Lausen, Switzerland), were obtained from AXXORA, LLC (San Diego, USA). In some experiments, mononuclear cells from SMGs, NPs and NALT were taken 5 days after the nasal immunization step and were stained with FITC-conjugated anti-CD11c and PE-labeled anti-MHC class II, -CD40, -CD80, or -CD86 mAbs (BD PharMingen). The samples were analyzed using a FACSCalibur (BD Biosciences) and CellQuest software (BD Immunocytometry Systems). In order to purify B-1 B cells, cells were stained with anti-B220 and anti-CD5 mAbs and were then subjected to FACSAria (DiVa 4.0) system (BD Biosciences).

### Cell separation by AutoMACS

To obtain mucosal DCs, mononuclear cells from SMGs, NPs and NALT were incubated with anti-CD11c labeled microbeads (Miltenyi Biotec Inc.) according to the manufacture's instructions and were then positively selected with an AutoMACS. The purified DC fraction consisted of >97% CD11c^+^, with >99% cell viability. Peritoneal IgA^−^ B cells were collected as follows. Mice were sacrificed, and the peritoneal cavity was gently washed with 5 ml sterile PBS. The peritoneal cells in the washed PBS were incubated with biotin-conjugated anti-IgA mAb, followed by streptavidin-conjugated microbeads, and IgA^−^ cells were negatively sorted using AutoMACS. Subsequently, the IgA^−^ cells were incubated with anti-B220 mAb-conjugated microbeads to isolate IgA^−^ B cells.

### Semi-quantitative and quantitative RT-PCR analyses

Total RNA was extracted from mucosal B-1 B cells using RNAeasy (Qiagen, Tokyo, Japan). The cDNA was prepared by Superscript III First-Strand Synthesis System (InVitrogen) with oligo(dT) primer. AID, Iα-Cμ (αCTs), Iμ-Cα, and β-actin transcripts in mucosal B-1 B cells were amplified by oligonucleotide primers specific for AID, αCTs, Iμ-Cα and β-actin transcripts as described previously [16], [33], [36], [37]. The RT-PCR products were analyzed on 2% agarose gels. The PCR product band densities of the respective CSR-associated molecules were calculated by the PCR product band density of β-actin expression as 100 with ChemiDoc XRS Quantity One Analysis software (Bio-Rad). In order to quantitate BAFF and APRIL expression on mucosal DCs and their receptor expression on B-1 B cells, the levels of synthesized cDNA of DCs and B-1 B cells from mucosal tissues were measured using a Nanodrop 2000 RNA/DNA calculator (Thremo Fisher Scientific Inc.). The sample cDNA and the external standards were amplified with a pair of specific primers for APRIL, BAFF, TACI, BCMA and BAFF-R in the presence of the Power SYBR Green PCR Master Mix (Applied Biosystems) by a Real-time PCR system (Applied Biosystems). The concentration of sample cDNA was determined using linear, diluted external standards obtained by an identical PCR protocol.

### Induction of in vitro IgA CSR by mucosal DCs

To examine the function of mucosal DCs for IgA B-1 B cell CSR, peritoneal IgA^−^ B cells were co-cultured with mucosal DCs from SMGs, NPs and cervical lymph nodes (CLNs) of mice given nasal TNP-LPS plus nCT, TNP-LPS alone or nCT only in the presence of IL-5, TGF-β_1_ or IL-5 and TGF-β_1_ cytokines. After stimulation for 5 days, nonadherent cells were harvested and were then stained with FITC-conjugated anti-IgA and PE-labeled anti-CD5 mAbs (BD PharMingen). The samples were then analyzed using the FACSCalibur® (BD Biosciences) and CellQuest software (BD Immunocytometry Systems).

### Statistical analysis

The results are expressed as the mean ± one standard error of the mean (SEM). All mouse groups were compared with control mice using an unpaired Mann-Whitney *U* test with Statview software (Abacus Concepts Inc.) designed for Macintosh computers. *P* values of <0.05 were considered significant.

## Results

### Induction of CSR-associated mRNA in oral mucosal effector tissues

We initially examined CSR from the μ-chain to the α-chain in B-1 B cells from SMGs and NPs as mucosal effector tissues of C57BL/6 mice. The mRNA levels specific for activation-induced cytidine deaminase (AID), αCTs and Iμ-Cα were analyzed by semi-quantitative RT-PCR. As positive controls, B-1 B cells from NALT of mice given nasal nCT and TNP-LPS were employed. Of importance, the levels of CSR-associated molecule-specific mRNA were increased in B-1 B cells from SMGs and NPs of mice given nasal TNP-LPS plus nCT when compared with those of mice given nasal TNP-LPS alone or nCT alone (upper section in Table 1). Although lower levels of AID- and αCTs-specific mRNA expression were seen in SMGs and NPs when compared with NALT, Iμ-Cα-specific mRNA transcripts in these mucosal effector tissues were essentially the same as those in NALT (Table 1). These findings indicate that higher levels of IgA CSR are induced in the oral-nasopharyngeal effector tissues in addition to NALT when C57BL/6 mice were nasally immunized with TNP-LPS plus nCT.

**Table 1:** Expression of AID, αCT, Iμ-Cα transcripts in B-1 B cells from the oral-nasopharyngeal effector and inductive tissues of mice nasally immunized with TNP-LPS with/without nCT or nCT alone.^a^^,^ b ^,^ c

Mouse	Tissue	AID			Iα-Cμ (αCT)			Iμ-Cα		
		nCT+TNP-LPS	TNP-LPS	nCT	nCT+TNP-LPS	TNP-LPS	nCT	nCT+TNP-LPS	TNP-LPS	nCT
C57BL/6	SMGs	* ^,^ # 67±5.4	23±3.4	18±3.3	* ^,^ # 36±5.5	13±4.0	9.2±1.8	* ^,^ # 72±6.9	11±2.9	26±6.2
	NPs	* ^,^ # 64±3.6	24±8.8	29±4.5	* ^,^ # 32±8.9	24±5.7	19±1.4	* ^,^ # 74±9.1	21±5.3	30±3.4
	NALT	* ^,^ # 84±9.2	25±6.8	37±5.4	* ^,^ # 41±7.4	28±3.6	28±4.5	* ^,^ # 78±11	19±4.3	32±4.8
TCRβ^−/−^	SMGs	* ^,^ # 39±7.0	20±3.7	21±2.7	* ^,^ # 26±8.4	7.8±4.2	9.9±5.1	* ^,^ # 60±16	8.1±3.0	11±2.2
	NPs	* ^,^ # 42±5.5	18±4.9	16±2.3	* ^,^ # 29±5.2	18±3.5	21±2.5	* ^,^ # 62±14	22±5.9	32±3.9
	NALT	* ^,^ # 62±8.8	16±6.9	25±3.4	* ^,^ # 45±9.4	19±2.0	16±1.9	* ^,^ # 66±15	28±6.6	30±4.1

a Five days after nasal immunization, B-1 B (CD5^+^ B220^+^) cells from the SMGs, NPs and NALT (as a positive control) were purified by FACS and were then subjected to semi-quantitative RT-PCR.

b The numbers are mean the expression percentages of the respective CSR-associated molecules when the density of β-actin expression on respective lymphoid tissues was calculated as 100 with ChemiDoc XRS Quantity one Analysis software (Bio-Rad).

c The values are presented as the mean ± SEM of 10 mice for each group and represent a total of five separate experiments.

*p<0.05 when compared with mice given TNP-LPS alone.

# *p*<0.05 when compared with mice given nCT alone.

### T cell-independent IgA CSR occurs in the SMGs and NPs

**Figure 1:**
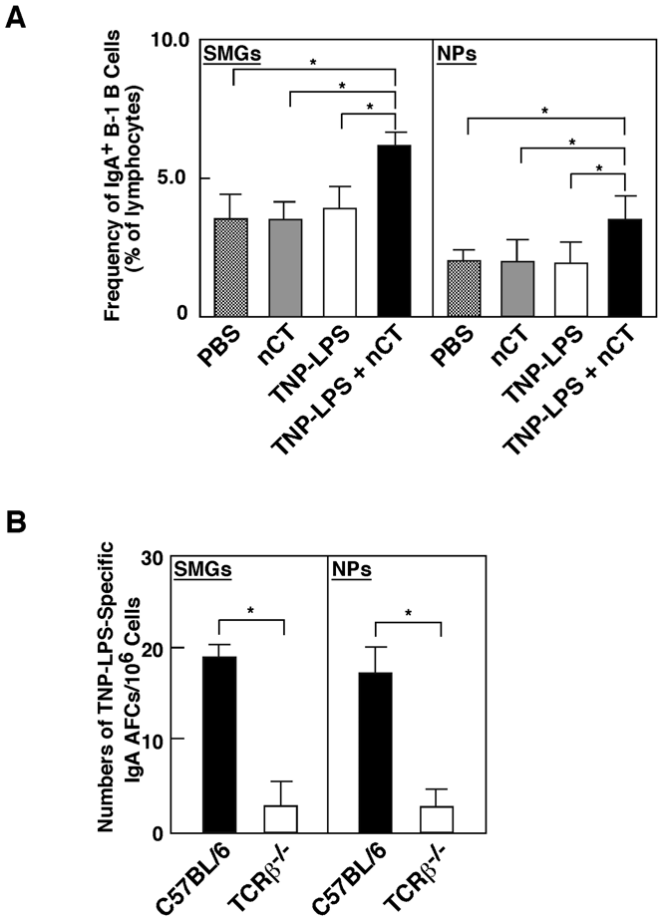
Frequencies of IgA^+^ B-1 B cells and TNP-LPS-specific IgA AFCs in the oral-nasopharyngeal effector tissues of TCRβ^−/−^mice. (A) TCRβ^−/−^ mice were nasally immunized with PBS alone, nCT alone or TNP-LPS (10 µg) with or without nCT (1 µg). Five days after nasal immunization, mononuclear cells were isolated from SMGs and NPs and were then stained with FITC-conjugated anti-IgA, PE-labeled anti-CD5, and APC-tagged anti-B220 mAbs. Samples were subjected to flow cytometry analysis by FACSCalibur®. *p<0.05 when compared with TCRβ^−/−^ mice given TNP-LPS alone, nCT alone or PBS. (B) Five days after TCRβ^−/−^ and their genetic background (C57BL/6) mice were nasally immunized with TNP-LPS (10 µg) plus nCT (1 µg), mononuclear cells isolated from SMGs and NPs of both groups of mice were subjected to a TNP-specific IgA AFC ELISPOT assay to determine numbers of IgA AFCs. *p<0.05 when compared with C57BL/6 mice. The values are presented as the mean ± SEM of 10 mice for each group and a total of five separate experiments.

Since nCT is known to be a potent Ag as well as adjuvant, it is possible that nasal CT enhances IgA CSR in oral-nasopharyngeal effector tissues through T cell help. In order to ensure that IgA CSR was induced in a T cell-independent (TI) manner, we analyzed the frequencies of IgA-expressing B-1 B cells and the expression of CSR-associated molecules in TCRβ-chain deficient (TCRβ^−/−^) mice. Significantly increased numbers of sIgA^+^ B-1 B cells were seen in the SMGs and NPs of TCRβ^−/−^ mice given nasal TNP-LPS plus nCT (Figure 1A). In addition, higher levels of AID, Iα-Cμ and Iμ-Cα-specific mRNA were detected in B-1 B cells from SMGs, NPs of TCRβ^−/−^ mice given nasal TNP-LPS plus nCT than were noted in TCRβ^−/−^ mice given nasal TNP-LPS only or nCT alone (lower section in Table 1). However, increased numbers of TNP-LPS-specific IgA AFCs were not seen in either SMGs or NPs of TCRβ^−/−^ mice given nasal TNP-LPS plus nCT when compared with those of similarly treated normal C57BL/6 mice (Figure 1B). Taken together, these results indicate that IgA CSR takes place in the oral-nasopharyngeal effector tissues in a TI manner. However, the differentiation to IgA-producing plasma cells may require CD4^+^ T cell help.

### Increased numbers of activated DCs in mucosal tissues

We next characterized CD11c^+^ DCs in SMGs, NPs and NALT, since DCs are known to play key roles in TI IgA CSR [24], [25]. Although numbers of CD11c^+^ DCs were unchanged in various mucosal lymphoid tissues of wild-type mice given nCT as nasal adjuvant (experimental group) when compared with wild-type mice given TNP-LPS alone (control group) (Table 2), significant up-regulation of co-stimulatory molecules was noted (Table 2). Thus, SMGs, NPs and NALT in the experimental group showed significantly elevated levels of CD80 and CD86 expression by CD11c^+^ DCs. Interestingly, only NALT DCs from the experimental group showed increased levels of CD40 expression, in addition to CD80 and CD86 (Table 2). These results indicate that nasal administration of nCT plus Ag induces activation of DCs in SMGs, NPs and NALT.

**Table 2:** The frequencies of CD11c^+^ DCs and co-stimulatory molecule expression in wild-type mice given nasal TNP-LPS with or without nCT^a^.

Tissue	Nasal Adjuvant	% of total lymphocytes	% of CD11c^+^ DCs			
		CD11c^a^	CD40^b^	CD80^b^	CD86^b^	MHC II^b^
SMGs	nCT	1.8±0.6	0.5±0.1	* 2.9±0.5	*20.9±4.9	50.2±8.8
	None	2.0±0.4	0.2±0.0	0.7±0.2	11.3±2.2	46.3±10.9
NPs	nCT	5.2±2.1	3.1±0.8	* 15.2±2.1	*7.9±1.3	18.0±4.2
	None	5.6±2.6	3.4±1.1	5.2±0.7	3.7±1.1	16.9±3.7
NALT	nCT	2.2±0.5	* 7.7±1.5	* 18.2±3.2	*17.2±2.8	50.6±12.2
	None	2.1±0.7	3.7±1.1	5.5±1.9	7.4±2.1	48.1±9.9

a Mononuclear cells from SMGs, NPs and NALT were isolated five days after immunization with TNP-LPS plus nCT or TNP-LPS alone, and were stained with FITC-conjugated anti-CD11c mAb.

b Mononuclear cells were stained with FITC-conjugated anti-CD11c and PE-labeled anti-CD40, -CD80, -CD-86 or -MHC class II mAbs and were then subjected to flow cytometry analysis by FACSCalibur®.

*p<0.05 compared with immunized mice given TNP-LPS alone.

### Nasal TNP-LPS plus nCT increases APRIL expression by DCs in SMGs, NPs and NALT of wild-type mice

**Figure 2:**
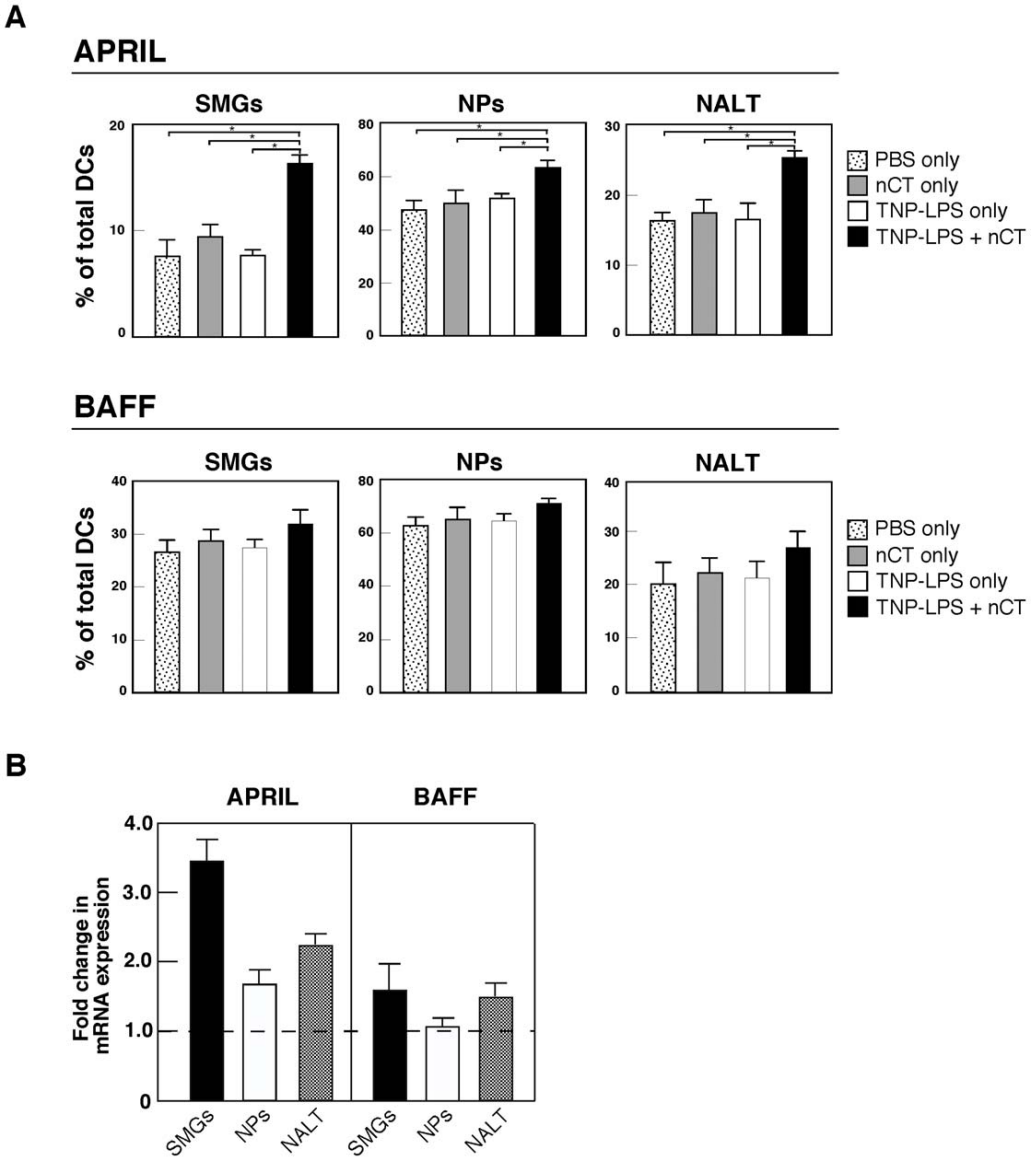
APRIL and BAFF expression by CD11c^+^ DCs. Mice were nasally immunized with TNP-LPS with or without nCT, nCT alone or PBS. (A) Five days after immunization, mononuclear cells were isolated from SMGs, NPs and NALT and stained for CD11c, APRIL and BAFF using the respective fluorescence-conjugated mAbs described in the Materials and Methods section. Samples were subjected to flow cytometric analysis by FACSCalibur®. *p<0.05 when compared with mice given TNP-LPS or nCT alone. The values are presented as the mean ± SEM of 10 mice for each group and represent a total of five separate experiments. (B) CD11c^+^ DCs were isolated from SMGs, NPs and NALT of mice given nasal TNP-LPS with or without nCT by AutoMACS two days after the immunization. The expression of BAFF- and APRIL-specific mRNA by mucosal DCs was determined by quantitative real-time PCR. Relative expression of BAFF- and APRIL- specific mRNA were displayed as the fold change of respective transcript expression by experimental group (TNP-LPS plus nCT) over the expression by controls (TNP-LPS alone). The values are presented as the mean ± SEM of 10 mice for each group and represent a total of five separate experiments.

Since it has been reported that intestinal DCs induce TI IgA CSR through APRIL and BAFF molecules [21], we next examined whether nCT-activated DCs from SMGs and NPs also express these molecules. The frequencies of APRIL- and BAFF-expressing DCs were essentially the same among mice given PBS, TNP-LPS only or nCT only. In contrast, significantly higher frequencies of APRIL-expressing DCs were noted in SMGs, NPs and NALT of mice given nasal TNP-LPS plus nCT than in mice given nasal TNP-LPS or nCT alone (Figure 2A). Although the frequencies of BAFF-expressing DCs were increased when mice were also given nCT plus TNP-LPS, this increase was modest (Figure 2A). Thus, mRNA analyses showed similar patterns of APRIL- and BAFF-specific mRNA expression by DCs from SMGs, NPs and NALT (Figure 2B). These results suggest that APRIL-expressing NALT DCs most likely migrate into the mucosal effector tissues for the induction of IgA CSR specific for TI Ag.

### Nasal TNP-LPS plus nCT induces BAFF-R, TACI and BCMA expression by salivary gland-associated B-1 B cells

**Figure 3:**
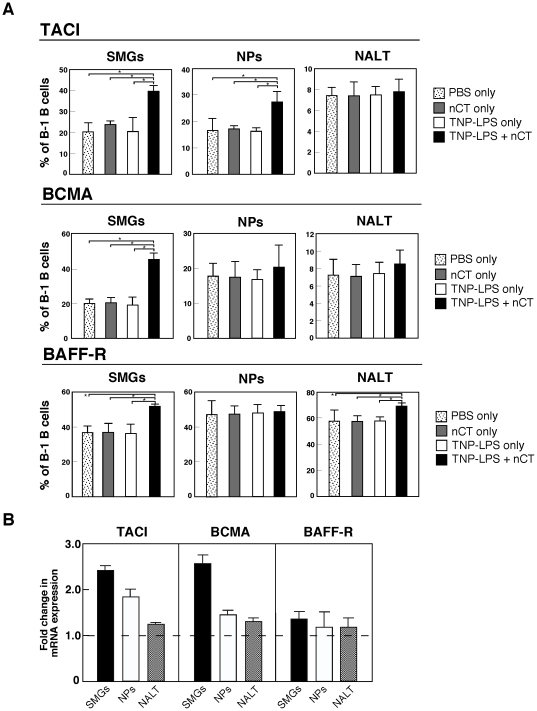
TACI, BCMA and BAFF-R expression by mucosal B-1 (CD5^+^ B220^+^) B cells. Wild-type mice were nasally immunized with TNP-LPS with or without nCT, nCT alone or PBS. (A) Five days after immunization, mononuclear cells were isolated and stained for CD5, B220, TACI, BCMA and BAFF using the respective fluorescence-conjugated mAbs as described in the Materials and Methods section. Samples were then subjected to flow cytometric analysis by FACSCalibur®. *p<0.05 when compared with mice given TNP-LPS or nCT alone. The values are presented as the mean ± SEM of 10 mice for each group and represent a total of five separate experiments. (B) B-1 B cells were purified by FACSAria from SMGs, NPs and NALT of mice given nasal TNP-LPS with or without nCT two days after the immunization. The expression of TACI, BCMA and BAFF-R mRNA by B1-B cells was assessed by quantitative real-time PCR. Relative expression of TACI, BCMA and BAFF-R mRNA are displayed as the fold change of respective transcript expression by the experimental group (TNP-LPS plus nCT) over the expression by controls (TNP-LPS alone). The values are presented as the mean ± SEM of 10 mice for each group and represent a total of five separate experiments.

We next examined the expression of receptors for APRIL and BAFF molecules by B-1 B cells from SMGs, NPs and NALT of wild-type mice given TNP-LPS nasally with or without nCT, nCT alone or PBS. The frequencies of TACI-expressing B-1 B cells were markedly increased in SMGs and NPs when mice were nasally immunized with TNP-LPS plus nCT (Figure 3A). In addition, SMGs showed increased numbers of BAFF-R- and BCMA-expressing B-1 B cells. These findings suggest that molecular interactions between DCs and B-1 B cells through APRIL and their receptors, such as TACI, play a key role in TI IgA CSR in oral-nasopharyngeal effector tissues. On the other hand, mucosal inductive tissues such as NALT contain a relatively small frequency of CD5^+^ B220^+^ B cells and the majority of these cells express BAFF-R when mice were nasally immunized with TNP-LPS, nCT or TNP-LPS plus nCT (Figure 3A). When nCT was employed as nasal adjuvant with TI-Ag, BAFF-R but not TACI or BCMA expression by B-1 B cells was significantly increased (Figure 3A). These findings indicate that NALT B-1 B cells are not the source of B-1 B cells in the oral-nasopharyngeal effector tissues for TI IgA CSR. In support of our FACS results, increased levels of TACI- and BCMA-specific mRNA were detected in SMGs of mice given nasal nCT plus TNP-LPS when compared with mice given nasal TNP-LPS only (Figure 3B). Further, significantly elevated levels of BCMA-specific mRNA were seen in B-1 B cells from NPs of mice given TNP-LPS plus nCT as nasal adjuvant (Fig. 3B). In contrast, essentially no up-regulation of TACI-, BCMA- or BAFF-R gene expression was detected in B-1 B cells from NALT (Figure 3B).

### Mucosal DCs induce IgA CSR by B-1 B cells in vivo

**Figure 4:**
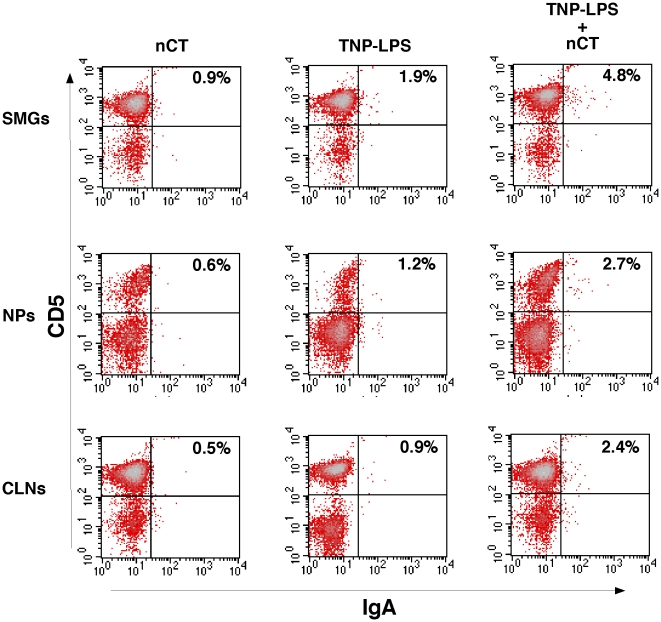
Induction of IgA CSR by B-1 B cells in the presence of mucosal DCs. IgA negative B cells were purified from the peritoneal cavity of wild-type mice by magnetic sorting. Purified cells (8×10^6^ cells/ml) were then cultured with DCs (4×10^5^ cells/ml) from SMGs, NPs and CLNs of mice given nasal TNP-LPS with/without nCT or nCT alone in the presence of 1 ng/ml TGF-β_1_, and 100 ng/ml IL-5. After 5 days of incubation, non-adherent cells were harvested and stained with FITC-conjugated anti-IgA, and PE-labeled anti-CD5 mAbs. The data represent a typical profile of 10 mice for each group.

To further examine whether NALT-derived DCs directly induce TI IgA CSR in B-1 B cells, CD11c^+^ DCs isolated from SMGs, NPs and CLNs of mice nasally immunized with TNP-LPS with/without nCT or nCT alone were cultured with peritoneal IgA^−^, IgM^+^ B cells in the presence of TGF-β_1_, IL-5 or both TGF-β_1_ and IL-5. Five days after incubation, non-adherent cells were isolated and subjected to flow cytometry analysis in order to determine the frequency of IgA and CD5 expressing B cells. When adding TGF-β_1_ alone or IL-5 alone, increased frequencies of IgA^+^ B-1 B cells were not seen in every mice group (Table 3). However, in the presence of both TGF-β_1_ and IL-5, higher frequencies of IgA^+^ B-1 B cells were seen in cultures containing CD11c^+^ DCs from SMGs, NPs and CLNs of mice nasally treated with TNP-LPS plus nCT when compared with those cultures incubated with DCs from mice given TNP-LPS alone or nCT only (Figure 4 and Table 3). These findings clearly showed that DCs in mucosal effector tissues play key roles in the induction of B-1 B cell IgA CSR in the presence of IL-5 and TGF-β_1_. Induction of IgA CSR by CLN DCs also indicates that DCs in oral-nasopharyngeal effector tissues potentially originate from NALT and CLNs since CLNs drain not only NALT cells but also nasal Ag and adjuvant.

**Table 3:** Induction of IgA CSR on B-1 B cells co-cultured with DCs plus cytokines^a^.

Nasal Immunization with	DCs from (4×10^5^ cells/ml)	% of IgA+ cells on B-1 B cells		
		IL-5 (100 ng/ml)	TGF-β_1_ (1 ng/ml)	IL-5 (100 ng/ml)+TGF-β_1_ (1 ng/ml)
nCT	SMGs	0.5±0.2	0.2±0.2	0.8±0.3
	NPs	0.4±0.2	0.2±0.3	0.7±0.2
	CLNs	0.2±0.3	0.0±0.2	0.5±0.2
TNP-LPS	SMGs	0.4±0.4	0.2±0.8	1.9±0.8
	NPs	0.3±0.2	0.3±0.2	1.2±0.4
	CLNs	0.4±0.5	0.2±2.6	0.9±0.3
TNP-LPS + nCT	SMGs	0.5±0.1	0.3±0.1	* ^,^ #4.8±0.5
	NPs	0.4±0.2	0.4±0.2	* ^,^ #2.7±0.7
	CLNs	0.4±0.3	0.4±0.1	* ^,^ #2.4±0.7

a IgA negative B cells from SMGs, NPs and CLNs from the peritoneal cavity cells were co-cultured with DCs plus IL-5, TGF-β_1_, or both IL-5 and TGF-β_1_ for five days, and were stained with FITC-conjugated anti-IgA mAb and were then subjected to flow cytometry analysis by FACSCalibur®.

*p<0.05 compared with immunized mice given TNP-LPS alone.

# *p*<0.05 compared with immunized mice given nCT alone.

## Discussion

We initially investigated the expression of AID, αCT and Iμ-Cα molecules involved in the induction of IgA CSR by B-1 B cells from SMGs and NPs of mice given nCT plus TNP-LPS, since it has been shown that AID is essential for CSR which specifically occurs in activated B cells [18], [21], [23]. Furthermore, αCT and Iμ-Cα transcripts are known as a hallmark to demonstrate active CSR *in vivo* [37]. As positive controls, the elevated mRNA expression of these IgA CSR-associated molecules were prominently seen in B-1 B cells from NALT of mice given TNP-LPS plus nCT. Interestingly, we also noted higher expression of IgA CSR-associated molecules by B-1 B cells in SMGs and NPs when compared with those from mice given nasal TNP-LPS or nCT alone. These results clearly show that TI IgA CSR takes place in the oral-nasopharyngeal effector tissues. Indeed, it has been well accepted that a TI IgA CSR pathway exists in the intestinal mucosa in addition to the classical T cell-dependent one [38]. Thus, intestinal IgA Abs are produced by B-1 B cells in diffuse effector lymphoid tissues such as intestinal lamina propria and are derived from the peritoneal cavity [31]. Despite these reports, our results are the first to show that up-regulation of the TI μ to α isotype CSR by B-1 B cells occurs in the oral-nasopharyngeal effector tissues in addition to the intestinal effector tissues when nCT was employed with TI-Ag as nasal adjuvant.

Since nasal nCT possesses strong adjuvant activity to enhance CD4^+^ T cell function [11], it was possible that the enhancement of IgA CSR may be induced by activated CD4^+^ T cells and their derived cytokines. Our previous results clearly showed that increased numbers of IgA^+^ B-1 B cells in both SMGs and NPs was only noted in mice given nasal TNP-LPS plus nCT, but not from mice given nasal TNP-LPS alone [34]. To eliminate the potential role for nCT-activated CD4^+^ T cells, TCRβ deficient (TCRβ^−/−^) mice were nasally immunized with TNP-LPS plus nCT. Our results revealed that AID, αCT and Iμ-Cα transcripts were upregulated in SMGs and NPs of TCRβ^−/−^ mice given nasal TNP-LPS plus nCT as mucosal adjuvant. To this end, the frequencies of IgA^+^ B-1 B cells and expression levels of IgA CSR-associated molecules in TCRβ^−/−^ mice were essentially the same as those detected in C57BL/6 mice (Table 1 and Fig. 1). Interestingly, TCRβ^−/−^ mice given nasal nCT showed reduced numbers of TNP-LPS-specific IgA AFCs in both SMGs and NPs when compared with those of normal controls given nasal TNP-LPS plus nCT. These results agree with our previous finding that CD4^+^ T cell-derived IL-5 is essential for TNP-LPS-specific IgA Ab synthesis [34]. Taken together, induction of IgA CSR by B-1 B cells, but reduced TNP-LPS-specific IgA AFCs in TCRβ^−/−^ mice, clearly indicate that nasal nCT plus TI-Ag enhances IgA CSR in SMGs and NPs in a T cell-independent manner; however, the subsequent events for the final differentiation of IgA^+^ B cells to IgA-producing plasma cells requires IL-5-producing CD4^+^ T cells.

It has been reported that both intestinal DCs and epithelial cells induce CD40-independent IgA CSR through BAFF and APRIL molecules [18], [21]. However, it still remains unclear whether DCs in oral-nasopharyngeal mucosal effector tissues express these molecules and serve a similar function as intestinal DCs. In order to shed light on the cellular and molecular mechanisms of TI IgA CSR in SMGs and NPs, we focused on DCs in SMGs and NPs for their potential roles in the induction of TI μ to α isotype CSR. Our results clearly showed increased numbers of APRIL-expressing CD11c^+^ DCs in SMGs and NPs of mice given TNP-LPS plus nCT when compared with those expressed by CD11c^+^ DCs from mice given nasal TNP-LPS or nCT alone. Further, when DCs from SMGs and NPs of mice given nasal TNP-LPS plus nCT were co-cultured with IgA^−^ B cells from the peritoneal cavity in the presence of both IL-5 and TGF-β_1_, high numbers of IgA^+^ B-1 B cells were induced. On the other hand, cultures containing DCs from SMGs or NPs of mice given nasal TNP-LPS or nCT alone revealed essentially the same frequencies of IgA^+^ B-1 B cells as seen in cultures containing B cells only (data not shown). These results clearly showed that APRIL-expressing DCs play a key role in IgA CSR by B-1 B cells in oral-nasopharyngeal effector tissues such as SMGs and NPs. It was recently reported that intestinal DCs activated through TLR5 signaling produce retinoic acid (RA) and induce IgA Ab production by peritoneal B-1 B cells [39]. Thus, it is possible that nasal nCT-stimulated DCs in SMGs and NPs produce RA for the induction of TNP-LPS-specific S-IgA Ab responses. Indeed, our results revealed increased levels of CD40, CD80, CD86 and MHC class II expression by DCs isolated from SMGs and NPs of mice given TNP-LPS plus nCT. However, nasal nCT failed to upregulate levels of retinal dehydrogenase (which converts retinal into retinoic acid)-specific mRNA expression by oral-nasopharyngeal DCs (data not shown). These results agree with our previous finding that nasal nCT-induced IL-5 synthesis by mucosal CD4^+^ T cells is essential for the induction of TNP-LPS-specific S-IgA Abs [34]. Taken together, our present study indicates that nasal nCT stimulated DCs in SMGs and NPs play a central role in IgA CSR, but not in the induction of terminal B cell differentiation into IgA-producing plasma cells.

B-1 B cells, known to be a minor subset comprising ∼5% of the total B cell population in the mouse and human periphery, arise during fetal development and have a restricted B cell receptor repertoire [40]. It has been previously reported that two distinct lineages of sIgA**^+^** B cells developed from B-1 and B-2 B cells and both, are involved in the induction of S-IgA Abs for mucosal immunity [41], [42]. In addition, peritoneal B-1 B cells and splenic marginal zone B cells play a central role in the induction of TI IgA Ab responses [31], [32]. Importantly, it has been reported that B-1 B cells express receptors for BAFF and APRIL-BAFF-R and TACI, respectively [43]. Since BAFF and APRIL are able to interact with their potential receptors such as BAFF receptor, BCMA and TACI molecules, it was important to identify which receptors on B-1 B cells in oral-nasopharyngeal effector tissues are responsible for the induction of IgA CSR. Higher frequencies of TACI-expressing B-1 B cells were detected in SMGs and NPs of mice given nasal nCT plus TNP-LPS than those cells from mice given nasal TNP-LPS only. These results agree with previous reports that APRIL promotes T cell-independent IgA CSR through engagement of TACI *in vitro* [21], . Further, it has been shown that APRIL on DCs interacts with BCMA and TACI on B cells for the induction of IgA CSR [44]. In this regard, SMG B-1 B cells also showed increased numbers of BCMA-expressing B-1 B cells when mice were nasally immunized with TNP-LPS plus nCT. Taken together, these findings suggest that molecular interactions between APRIL-expressing DCs and TACI- or BCMA-bearing B-1 B cells play a major role in T cell independent IgA CSR in SMGs and NPs.

In summary, although it was evident that TI IgA isotype class switching occurs in diffuse mucosal effector tissues such as the intestinal lamina propria, our present study clearly showed that this event also takes place in oral-nasopharyngeal effector tissues. Further, nasal nCT as mucosal adjuvant enhanced IgA CSR in the SMGs and NPs for subsequent S-IgA Ab production. Thus, CD11c^+^ DCs from SMGs and NPs induce T cell independent IgA CSR by sIgM^+^ IgA**^−^** B-1 B cells through interactions between APRIL and TACI/BCMA molecules. However, T cell deficiency abolished enhancement of TNP-LPS-specific S-IgA Ab responses by nCT. Taken together, the nasal adjuvant effect of nCT in TI-Ag-specific IgA Ab responses were uniquely regulated by TI IgA CSR by DC-B-1 B cell interactions. In the present study, we have elucidated a novel cellular and molecular mechanism for nCT adjuvanticity for the induction of TI-Ag-specific immunity in the oral-nasopharyngeal tract. It has yet to be shown that nCT serves a similar function in the GI tract. This will be elucidated in our future studies.
